# Efficacy of a Virtual Reality Game on Children’s Fear and Anxiety During Dental Procedures (VR-TOOTH): Protocol for a Randomized Controlled Trial

**DOI:** 10.2196/83672

**Published:** 2026-01-29

**Authors:** Julien Gardner, Vallerie Markopoulos, Wenjia Wu, Gabrielle Gilbert, Daphnée Pelletier, Charlotte Fafard, Anne Gagné, Estelle Guingo, Christine Genest, Marie-Ève Asselin, Pascale Ouimet, Kate St-Arneault, Sylvie Le May

**Affiliations:** 1 Faculty of Medicine, Université de Montréal, Montreal, QC, Canada; 2 Research Center, Centre Hospitalier Universitaire Sainte-Justine, Montreal, QC, Canada; 3 Faculty of Dental Medicine, Université de Montréal, Montreal, QC, Canada; 4 Department of Dental Medicine, Centre Hospitalier Universitaire Sainte-Justine, Montréal, QC, Canada; 5 Faculty of Arts and Sciences, Univeristé de Montréal, Montreal, QC, Canada; 6 Université du Québec en Abitibi-Témiscamingue, Abitibi-Témiscamingue, QC, Canada; 7 Trauma Studies Centre, Institut Universitaire en Santé Mentale de Montréal, Montréal, QC, Canada; 8 Faculty of Nursing, Université de Montréal, Pavillon Marguerite-d'Youville, 2375 chemin de la Côte-Sainte-Catherine, Montreal, QC, H3T 1A8, Canada, 1 514 343-6384

**Keywords:** virtual reality, dentistry, paediatrics, pain, special health care needs, anxiety

## Abstract

**Background:**

Dental fear and anxiety (DFA) affects approximately a quarter of children and adolescents. It significantly contributes to pediatric patients avoiding dental care later in adulthood. Lack of cooperation due to DFA can create a stressful environment, often forcing dentists to end appointments prematurely and consider alternative pharmacological treatments. The use of virtual reality (VR) during dental procedures, offering an immersive sensory experience, may serve as an additional nonpharmacologic tool to better manage DFA in children with special health care needs (SHCN) undergoing dental treatment.

**Objective:**

This study aims to assess the effectiveness of VR immersion in reducing anxiety and pain among pediatric patients with SHCN undergoing dental procedures. The study also seeks to understand the satisfaction of parents and health care providers with the use of VR during dental appointments.

**Methods:**

This randomized controlled trial follows a parallel design with two groups: a control group receiving standard care and an experimental group using VR. A sample size of 400 participants was calculated. Participants will be randomly assigned equally to each group. Recruitment will take place at the dental clinic of the Centre Hospitalier Universitaire Sainte-Justine, a tertiary- and quaternary-care center that primarily serves pediatric patients with SHCN. The two primary outcomes will include both observed and objective biomarker-based measures of anxiety. DFA will be evaluated using the Venham Anxiety Rating Scale as well as changes in mean levels of salivary alpha-amylase. Sociodemographic characteristics, parents’ and health care professionals’ satisfaction levels, participants’ pain intensity and behavior during the procedure, changes in heart rate, occurrence of side effects, procedure duration, and any deviations from normal procedural length will also be collected. Descriptive and comparative statistics will be conducted for demographic and clinical comparisons and will be used to present sociodemographic and clinical data, parents’ and health care professionals’ satisfaction levels, child satisfaction with the game, and procedural time.

**Results:**

This study will be conducted from November 2023 to December 2025. As of November 2025, 300 participants have been recruited. Results are expected to be available in June 2026.

**Conclusions:**

We believe that the results of this study will confirm the efficacy of VR in reducing DFA in children with SHCN, providing an additional nonpharmacological alternative for better managing this condition in pediatric hospital settings.

## Introduction

### Background

Dental fear and anxiety (DFA) is defined as a patient’s psychological state of worry, anxiety, or even dread that is specific to a dental procedure [1]. Although different, dental fear and dental anxiety, which respectively represent an emotional response and a heightened state of apprehension to potential distress from dental treatment, are challenging to distinguish in clinical practice [2,3]. Consequently, the term “dental fear and anxiety” is frequently used to describe these intense negative emotional states associated with dental procedures. Children’s DFA disorder refers to the clinical manifestations of dental phobia or anxiety in children, including reduced cooperation, lessened compliance, and even resistance to treatment [4,5]. DFA are conditions that affect approximately 13.3% to 29.3% of children and adolescents and are a significant reason why patients avoid dental care [3,6]. In children, DFA is also associated with lower oral health-related quality of life [7]. Although the etiology of DFA is multifactorial, often stemming from both exogenous and endogenous sources, a past traumatic dental experience is the most predictive factor for DFA [8,9]. A study by Ten Berge et al [10] states that the majority of DFAs experienced by adults originates from poor dental experiences during childhood. These findings highlight the importance of making each dental visit a positive experience for pediatric patients. Short-term distress during appointments, if not managed appropriately, can accumulate into poor dental experiences and consequently, reinforce DFA into adulthood [11]. The long-term effects of these negative childhood dental experiences can lead patients to avoid seeking proper dental care in the future [12].

Pediatric dental patients with special health care needs (SHCN), such as patients with cancer, neurodivergent patients, or those with chronic conditions, including cardiovascular or pulmonary conditions or neurological afflictions, require extra time and special considerations during treatment due to medical, physical, cognitive, or developmental conditions [13]. This population includes children with behavior disorders (eg, autism spectrum, anxiety, and attention-deficit/hyperactivity disorder), congenital conditions (eg, trisomy 21 or congenital heart disease), developmental disorders (eg, cerebral palsy), systemic illnesses (eg, childhood cancer or sickle cell disease), or cognitive disabilities (eg, intellectual disability) [13]. Children with SHCN face more barriers to dental care than the overall population [13], including external factors like transportation, cost, inadequate dental facilities, as well as internal factors such as fear and poor tolerance [13,14]. Many children with SHCN are cared for by dentists in community settings or, more often, in hospital dental clinics [13]. They tend to experience a higher level of DFA, which can make dental visits more challenging [13]. Providing comprehensive care and ensuring each dental visit is positive for pediatric patients with SHCN is crucial in promoting good oral health habits and improving their oral health as they transition into adulthood [13]. Understanding and assessing DFA in children is key to delivering successful dental care with high satisfaction in this age group. Among the vast array of assessment methods available, self-report, parental proxy, observation-based, and physiological assessments are the four major types used to evaluate DFA in children [15]. Recently, salivary alpha-amylase, an enzyme linked to both adrenaline and noradrenaline, has also been used as a marker for autonomic nervous system activity and stress [16].

Factors in the dental setting that trigger DFA include loud dental instrument sounds, the presence of unfamiliar people examining the oral cavity, and fear of pain [17]. Of these, the biggest trigger of DFA is the anticipation and use of local anesthesia injections [9,17]. Although necessary for effective pain control during certain procedures, local anesthesia injections are understandably uncomfortable [9]. For children, the initial injection combined with the feeling of numbness during the procedure can be especially distressing [9]. Pharmacological agents, combined with light to moderate sedation or general anesthesia, can also be considered for noncooperative patients, who are often time-consuming and more costly and carry higher health risks [3]. In current research, audiovisual distractions such as tablets and TV screens have been used as additional distraction techniques beyond traditional tell-show-do methods, yielding overall positive results [18]. However, these techniques lack interactivity, resulting in less immersive distraction environments for children [19]. The lack of cooperation due to DFA often forces dentists treating pediatric patients to end appointments prematurely, sometimes without completing the planned procedures. Treating anxious and fearful patients can create a stressful environment for the clinician and the dental team [3]. Particularly when treating children with SHCN, extra time and tools are needed to ensure comfortable dental care. Depending on the child’s diagnosis, some may experience hypersensitivity to external stimuli such as loud noises, aversion to specific tastes, or difficulty straying from usual daily routines [20]. Furthermore, Pagano et al [20] showed that augmented reality was well suited for patients with autism spectrum disorder as preparation for their dental visits.

Additionally, a systematic review by Cunningham et al [21] concluded that virtual reality (VR) is a promising tool in dentistry, especially for children with autism spectrum disorder or other special health care needs. VR is defined as an artificial environment experienced through sensory stimuli [22]. It is a modern tool that immerses patients in a “game” or “virtual world.” Commonly used in medicine to distract patients during unpleasant procedures such as vaccination, cast removal, and short bedside interventions, VR has been proven effective at decreasing procedural anxiety and creating a more positive patient experience [23]. Limited existing research on VR in dentistry shows positive results for anxiety management during dental procedures. A recent clinical trial by Alshatrat et al [19] found that VR was effective in reducing anxiety in young children during dental procedures.

Additionally, a previous study by Ram et al [24] reported high satisfaction among both parents and clinicians using audiovisual glasses to guide children during dental treatments. However, research on clinical VR applications in pediatric dentistry remains limited, especially within populations with SHCN. VR offers potential as an additional nonpharmacologic tool for pediatric dentists. A clinical study assessing VR’s effectiveness during dental appointments in pediatric patients with SHCN could lead to better control of DFA and possibly facilitate easier procedures.

### The Study

#### Aims

The study aims to evaluate the efficacy of VR immersion as a tool in reducing anxiety in pediatric patients with SHCN undergoing dental procedures. The study also aims to gain insight into parents’ and health care providers’ satisfaction levels with the use of VR by children during dental treatments.

#### Hypothesis

We hypothesize that VR immersion will more effectively reduce anxiety in pediatric patients undergoing dental procedures than the standard intervention of watching a cartoon on a muted mounted TV set or tablet.

### Objectives

#### Primary Outcomes

The primary outcomes of this study are to measure both observed and objective biomarker-based indicators associated with DFA. The observed indicator will be evaluated using the Venham Anxiety Rating Scale (VARS) [25] (Table 1) and will be assessed by examining changes in mean levels of salivary alpha-amylase (SAA) between baseline (T0) and the middle of the intervention (T1) [26].

**Table 1.** The Venham Anxiety Rating Scale.

Rating	Definition
0	Relaxed, smiling, willing, and able to converse.
1	Uneasy and concerned. During a stressful procedure, the child may protest briefly and quietly to indicate discomfort. Hands remain down or partially raised to signal discomfort. Child willing and able to interpret experience as requested. Tense facial expression, may have tears in eyes.
2	Child appears scared. Tone of voice and questions and answers reflect anxiety. During a stressful procedure, verbal protest, crying or quiet crying, hands tense and raised (not interfering much—may touch the dentist’s hand or instrument, but not pull at it). The child interprets the situation with reasonable accuracy and continues to work to cope with his or her anxiety.
3	Shows reluctance to enter the situation, difficulty in correctly assessing situational threat. Pronounced verbal protest and crying. Uses hands to try to stop procedure. Protest out of proportion to threat. Copes with the situation with great reluctance.
4	Anxiety interferes with the ability to assess the situation. General crying not related to treatment. More prominent body movements. The child can be reached through verbal communication, and eventually, with reluctance and great effort, he or she begins the work of coping with the threat.
5	The child is out of contact with the reality of the threat. General loud crying, unable to listen to verbal communication, and makes no effort to cope with the threat. Actively involved in escape behavior. Physical restraint required.

#### Secondary Outcomes

The secondary outcomes of this study are to compare the following indicators between the VR distraction and the standard intervention groups:

1. Parents’ (Multimedia Appendix 1) and health care professionals’ (Multimedia Appendix 2) satisfaction levels
2. Child satisfaction with the use of VR (Multimedia Appendix 1).
3. Physiological parameters such as heart rate and oxygen saturation
4. Behavior during the procedure (Table 2)
5. Occurrence of side effects
6. Length of the dental procedure
7. Child’s pain intensity (Multimedia Appendix 3)

**Table 2.** The Venham Behavioral Rating Scale.

Rating	Definition
0	Total cooperation, best possible working conditions, and no crying or physical protest.
1	Mild or soft verbal protest, crying or quiet crying as a signal of discomfort, but does not obstruct progress. Appropriate behavior for the procedure, that is, slight start at injection, “ow” during drilling if hurting, and so on.
2	Protest more prominent. Both crying and hand signals. May move the head around making it hard to administer treatment. Protest is more distracting and troublesome. However, the child still complies with the request to cooperate.
3	Protest presents a real problem to the dentist. Complies with demands reluctantly, requiring extra effort by dentist. Body movement.
4	Protest disrupts procedure and requires that all of the dentist’s attention be directed toward the child’s behavior. Compliance was eventually achieved after considerable effort by the dentist, but without much actual physical restraint (may require holding the child’s hands or the like to start). More prominent body movement.
5	General protest, no compliance or cooperation. Physical restraint is required.

## Methods

### Design

This randomized controlled trial (RCT) will follow a parallel design with two groups: a control group (watching cartoons on a muted wall-mounted TV or a tablet) and an experimental group (VR intervention during the dental procedure).

### Sample and Setting

In mid-2023, we conducted a pilot study to evaluate feasibility and identify recruitment challenges before moving forward with the full RCT [27]. Between June 6, 2023, and July 20, 2023, 25 patients were recruited over 44 days at a single research site. Among these 25 patients, the mean VARS and Venham Behavioral Rating Scale scores before the procedure were 0.08 (SD = 0.28) for both scales, and during the procedure, the mean scores were 0.625 (SD = 1.35) and 0.667 (SD = 1.37), respectively. The variability in scores was higher at the time point with the higher average scores; therefore, we assumed a test with unequal variances. For the SAA outcome, data from a separate RCT conducted by our group were used [28]. This study measured changes in SAA before and during magnetic resonance imaging in children assigned to either a VR or a control condition. In that RCT, the mean difference in log-transformed SAA was 0.171 (SD = 1.05) in the control group and −0.096 (SD = 0.495) in the VR group. Because the expected difference in log SAA was smaller, this outcome guided our sample size calculations.

Therefore, to achieve 80% power to detect a mean difference of −0.096 (SD = 0.495) in log SAA using a 2-sided, 2-sample *t* test with unequal variances and a type-I error (α) of .05, a sample size of 364 participants (n=182 per group) was needed. Given the two primary outcomes, a Bonferroni correction was applied, reducing the α to .025. Based on an estimated 10% attrition rate from our pilot study, the final sample size was increased to 400 participants. Patients are currently being recruited for this study.

Recruitment is taking place at the dental clinic of the Centre Hospitalier Universitaire Sainte-Justine, a pediatric hospital in Montréal, Canada. This clinic mainly serves patients with SHCN, such as craniofacial abnormalities, autism spectrum disorder, or children battling cancer. Pediatric patients with SHCN make up about 80% of the clinic’s total clientele, while the remaining patients are otherwise healthy with dental trauma or other emergencies. The clinic sees roughly 5000 visits annually, including around 4000 patients with SHCN. Typically, about 600 (15%) of these patients have a severe diagnosis and are known to be completely uncooperative. Therefore, we estimate that approximately 3400 patients would be eligible for recruitment. Based on our pilot recruitment rate, we anticipate recruiting roughly 3 participants per week during September through April and 9 participants per week during May through August. Date collection is currently ongoing, and we expect to reach our target enrollment of 400 patients by the end of December 2025.

Participants will be identified by the clinic’s resident dentist using the scheduling system for upcoming appointments involving specific painful dental procedures, such as examinations, prophylaxis, extractions, and restorations. The research assistants will then contact the parents or legal guardians to provide information about the study and obtain their approval in advance. An independent individual, separate from the clinical dental team, will review the consent with both participants and parents. The information and consent form will be signed by a parent or legal guardian on the day of the visit. Whenever possible, patient assent will be sought.

### Inclusion Criteria

Children participating in this study must meet the following criteria: (1) be aged 6 to 17 years; (2) have received the dentist’s recommendation to participate; (3) need to undergo any dental procedure; and (4) be accompanied by a parent or legal guardian who can read, write, and understand either French or English.

### Exclusion Criteria

Participants will be excluded from this study if they (1) are diagnosed with epilepsy; (2) have paralysis or paresis of the hand; (3) have any diagnosed eye disease or problem; or (4) have any other conditions preventing them from using VR (eg, epidermolysis bullosa). Patients with a strong history of motion sickness will not be excluded from this study, but this information will be documented, and additional monitoring will be provided. The use of medication (opioid and nonopioid analgesics, antiemetics, anxiolytics, or any other drugs) prior to the procedure within the last 4 hours will not exclude participants from this study, but this information will be collected before the procedure and included as part of the sociodemographic and clinical information form. Patients who have received previous dental procedures at the recruiting setting will not be excluded.

### Randomization and Allocation

Randomization will be performed using the electronic REDCap (Research Electronic Data Capture; Vanderbilt University) system. Allocation to either intervention will be randomized by an independent biostatistician from the Unité de recherche clinique appliquée (Applied Clinical Research Unit). To equalize participants across both arms, permuted block randomization with randomly selected block sizes design will be used to randomize participants to their intervention [29]. Access to the randomization list will be granted only to the biostatistician, and allocation will be concealed in REDCap to reduce selection bias.

### Interventions

#### Control Intervention

Participants assigned to the control intervention will only receive the care-as-usual approach. This includes viewing cartoons on a muted, mounted television already set by the clinic, watching a video of their choice on a tablet on YouTube, and the use of pharmaceutical treatment during the procedure, such as injected local anesthesia, if indicated by the procedure. In the event of noncooperation during the appointment, any necessary procedural retakes or rescheduling of appointments as determined by the dentist will be documented by the research assistant. One parent will be permitted to be present in the room during the procedure as part of the clinic’s usual protocol, and their presence will be recorded. Children in the control intervention will be offered the option to try the VR game after the study period, if they choose to do so.

#### Experimental Intervention

**Figure 1.**
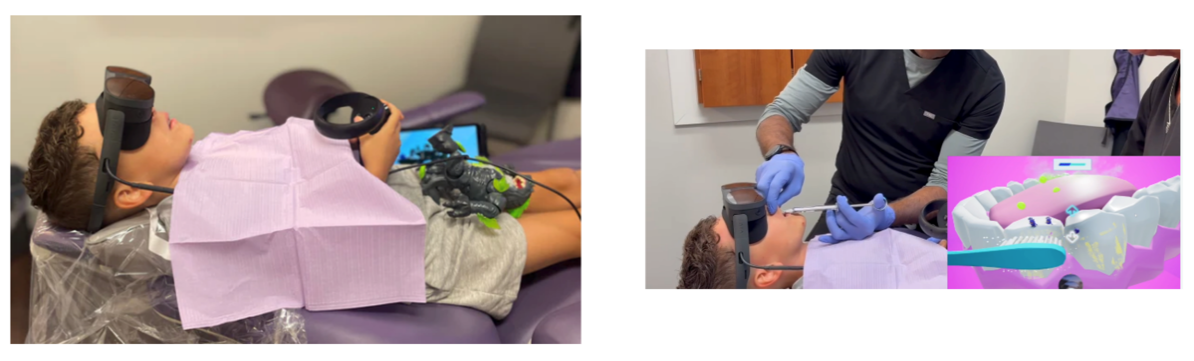
Patient’s position during a dental procedure and virtual reality accessory [30]. The images or screen captures were reproduced with permission from Paperplane Therapeutics.

Participants assigned to the experimental intervention will engage with a VR game called “Dream Dental,” tailored for a pediatric population. The simplified no-success game, developed by Paperplane Therapeutics [30], is enjoyable regardless of a child’s video game experience, and its point-and-shoot arcade style makes it easy to understand (Figure 1). It was designed specifically for this study, with the horizontal position required for dental procedures in mind (Figure 1). The VR video game aims to reduce anxiety in children aged 6 to 17 years by means of immersive distraction.

**Figure 2.**
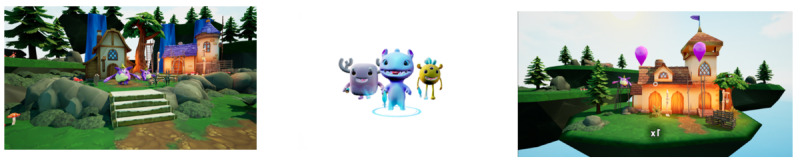
Dream Dental game environment [30].

We will use a lightweight head-mounted device (HMD; Pico Neo 4) with built-in eye tracking [31]. The Pico Neo 4 is a standalone VR headset featuring a 4K Super-Vision Display with pancake lenses for a clearer, broader field of view. Its key features include a balanced design with a rear-mounted battery, a high-resolution display (2160×2160 per eye), a 90 Hz refresh rate, and full-color pass-through via its 16 MP RGB camera. It also uses 6 degrees of freedom inside-out tracking and offers both wireless and wired PC VR connectivity. This headset was specifically designed to be lightweight and smaller to better fit children’s smaller heads. Children can play throughout the entire dental procedure using a clicker in one hand (Figure 1); eye-tracking sensors will assist navigation through the game, but will not be used for data collection. The VR game “Dream Dental” features a rail mechanism (predefined path or sequence of scenes to reduce body and head movement) that guides children through an interactive environment filled with entertaining characters. The game is designed for dental procedures (Figure 2) and incorporates innovative eye-tracking technology integrated into the headset. This technology simplifies navigation by enabling users to target objects with their eyes instead of relying on head movements. The goal is to make the game easier to use during dental procedures, where head movements need to be restricted. These features are also intended to reduce virtual motion sickness. The VR HMD allows children to view the game they are playing in real time while blocking their view of the procedure, and it was determined not to hinder dental procedures [27].

Pharmaceutical treatment during the procedure, such as injected local anesthesia, will be used as indicated. In the event of noncooperation during the appointment, any procedural retake or rescheduled appointments will be noted by the research assistant or resident dentist. One parent will be allowed in the room during the dental procedure, and their presence will be recorded.

#### Study Time Periods

The study time periods are as follows and are detailed in Table 3. T0 corresponds to the time before the start of the intervention, T1 represents the time during or at the midpoint during the procedure, and T2 corresponds to the period 10 minutes after the intervention.

**Table 3.** Study time periods and outcomes.

Study time periods	Outcomes									
	Patients’ SDC^a^	VARS^b^ anxiety	SAA^c^	Parents’ and health care professionals’ satisfaction	Patients’ satisfaction	Patients’ physiological parameters	VBRS^d^ behavior	Side effects	Length of procedure	FLACC-R^e^
T0^f^	✓	✓	✓			✓	✓	✓		✓
T1^g^		✓				✓	✓			✓
T2^h^			✓	✓	✓	✓		✓	✓	

a SDC: sociodemographic characteristics.

b VARS: Venham Anxiety Rating Scale.

c SAA: salivary alpha-amylase.

d VBRS: Venham Behavioral Rating Scale.

e FLACC-R: Face, Legs, Activity, Cry, and Consolability—Revised.

f T0: before the procedure.

g T1: midpoint during the procedure.

h T2: 10 minutes after the procedure.

### Measures and Outcomes

#### Sociodemographic and Clinical Characteristics

Sociodemographic characteristics will be collected in the waiting room, after consent and prior to the intervention, by the parent or legal guardian present, including age, sex, ethnicity, and the procedure needed. Other clinical information will also include the participant’s clinical diagnosis and any medication taken within the last 4 hours (name, class, and posology) that could affect the study’s outcome. Participants will also be asked if they have taken any medication (name) for attention or hyperactivity to assess its potential impact on their ability to be distracted by VR.

#### Primary Outcomes

The two primary outcomes of this study are to measure both observed and objective biomarker-based indicators associated with DFA.

#### Measures of Primary Outcomes

The first primary outcome will evaluate DFA using the VARS (Table 1) [25]. The VARS is an observation-based assessment by proxy for DFA and is among the most frequent anxiety scoring instruments for DFA [25]. The scale has been used as an anxiety rating scale in other studies that evaluated the efficacy of VR distraction in the management of DFA [32,33]. The anxiety scale consists of one item, with six defined anxiety levels that range from 0, representing the child being relaxed, smiling, and willing to cooperate, to 5, representing the child being out of contact with the reality of the threat. The highest score represents the highest anxiety level [25]. A high degree of reliability was observed for this scale, even by untrained observers [25,34]. A research assistant will indicate a score from 0 to 5 on the anxiety scale at T0 and T1 (Table 3).

The second primary outcome will assess the mean difference in SAA levels between T0 and T2, 10 minutes after the dental procedure. This enzyme is linked to both adrenaline and noradrenaline and is a validated and reliable specific biomarker of autonomic nervous system activity. It has been proposed as an alternative biomarker for anxiety, over salivary cortisol, as it reaches peak levels within 10 minutes after a stressful procedure compared to cortisol, which requires a 30-minute delay [16,35]. Enzyme sampling has been performed in previous studies to evaluate DFA in children undergoing dental procedures [35-37]. This enzyme is considered an accurate and noninvasive procedure for assessing anxiety-related events such as dental examination, cleaning, restoration, or extraction in children [26,35]. Alpha-amylase sampling has been performed in previous studies to evaluate DFA for children undergoing dental procedures [35,38]. Salivary samples will be collected using a sterile dental cotton swab from under the tongue and stored immediately within a sterile vial in a research-specific on-site freezer at −80 degrees Celsius. The research assistant will collect the samples and take them to the laboratory. Results will be entered in REDCap for analysis purposes after the study.

#### Secondary Outcomes

The secondary outcomes include (1) parents’ and health care professionals’ satisfaction in both groups and children’s satisfaction in the VR intervention; (2) changes in physiological parameters, such as heart rate and oxygen saturation during and after the intervention compared to baseline; (3) behavior during the procedure; (4) occurrence of side effects (nausea, dizziness, and vomiting); (5) length of the procedure; and (6) the effectiveness of VR in reducing pain (Table 3).

#### Measures of Secondary Outcomes

The secondary outcomes will be measured as follows:

1. Parents’ or legal guardians’ satisfaction with the intervention will be assessed using a Numerical Rating Scale (0‐10, where 0 is very dissatisfied and 10 is very satisfied) and according to the recommended question by the Pediatric Initiatives on Methods, Measurement and Pain Assessment in Clinical Trials: “Considering anxiety relief, side effects, and emotional recovery, how satisfied were you with the intervention used to manage anxiety experienced by your child?” [39] (Multimedia Appendix 1).
2. Health care professionals’ satisfaction with the intervention will be assessed using a 6-question satisfaction questionnaire, with a range of scores from 1 to 4 (1=“Totally disagree,” 2=“Disagree,” 3=“Agree,” and 4=“Totally agree”; Multimedia Appendix 2).
3. Child satisfaction with VR will be assessed using a 0‐10 numerical rating scale (where 0 is very dissatisfied and 10 is very satisfied; Multimedia Appendix 1).
4. Physiological parameters (heart rate and oxygen saturation) will be measured using the COVIDIEN Nellcor pulse oxygen saturation meter, an approved and validated device.
5. The patient’s behavior during the procedure will be assessed by proxy via a research assistant using the Venham Behavioral Rating Scale at T0 and T1 (Table 3) [25,40]. This 6-point scale assesses children’s behavior and provides information about patients with disruptive behavior. The scale points are based on objective, and readily observable behaviors and classify children’s behavior into 6 categories ranging from 0 (total cooperation) to 5 (general protest, no compliance, or cooperation). This scale has demonstrated good interrater reliability and has proven to be a valuable instrument for assessing children’s behavioral responses to dental procedures [40].
6. Side effects will be collected in children in both groups from arrival on site until discharge, using a checklist of side effects commonly associated with VR, namely nausea, dizziness, and vomiting. Any of these side effects will be recorded along with the time they will occur.
7. The length of the procedure in minutes will be measured and recorded for each participant and compared between the two groups.
8. Pain will be assessed using the Face, Legs, Activity, Cry, and Consolability—Revised scale (FLACC-R) [41] (Multimedia Appendix 3). This questionnaire will serve to assess VR efficacy in reducing pain by comparing the mean pain level of both groups (experimental and control intervention) during the dental procedure (T1).

The FLACC-R scale (Multimedia Appendix 3) is a tool developed by Malviya et al [41] to assess pain in infants and young children who are unable to communicate their pain verbally. Health care professionals use this scale to evaluate pain in children who may not be able to express their discomfort in words. The FLACC-R scale consists of five categories, each rated on a scale of 0 to 2, with 0 indicating no pain and 2 indicating severe pain. The categories are as follows:

1. *Face* (F): this category assesses the child’s facial expressions. It looks at whether the child is relaxed, smiling, or showing pain-related facial expressions, such as frowning, grimacing, or jaw clenching. A score of 0 indicates a relaxed face, while a score of 2 indicates a highly distressed or painful expression.
2. *Legs* (L): this category considers whether the child’s legs are relaxed or whether there is increased movement, kicking, or tensing of the leg muscles. A score of 0 implies relaxed legs, while a score of 2 indicates significant leg movement due to pain.
3. *Activity* (A): this category looks at whether the child is calm and content or if they are restless, agitated, or unable to stay still. A score of 0 indicates calmness, while a score of 2 indicates high activity and restlessness due to pain*.*
4. *Cry* (C): this category assesses the quality of the child’s cry, considering whether the child is not crying, whimpering, or having a high-pitched, continuous cry. A score of 0 means no crying, while a score of 2 implies a high-pitched, continuous cry*.*
5. *Consolability* (C): the *consolability* considers whether the child can be comforted easily or if they remain inconsolable despite efforts to soothe them. A score of 0 suggests easy consolability, while a score of 2 indicates that the child cannot be consoled.

The total score on the FLACC scale is obtained by summing the scores across the 5 categories and ranges from 0 (no pain) to 10 (severe pain). The FLACC-R score will be evaluated at T0 (baseline) and at 10 minutes after the start of the procedure or at midprocedure for shorter dental treatments (T1).

### Study Proceedings Including Data Collection

Participants will be screened by a resident dentist or a research assistant via the appointment scheduling system for any dental procedure. Since DFA can arise from multiple procedures, such as simple cleaning to teeth extraction. A research assistant will review consent with participants and parents. Written consent by the parents or legal guardian—including the child’s assent (if possible)—will be obtained on arrival at the clinic, the day of the procedure, while completing the preprocedural questionnaire. After verifying that the participant meets all inclusion and exclusion criteria, the research assistant will log into REDCap only 10 minutes before the start of the intervention to minimize the risk of bias toward the intervention, obtain the group allocation, and then announce it to the participant. Because of the nature of VR, no blinding will be possible to staff, participants, parents, or legal guardians. Baseline data collection prior to the dental procedure, including a sociodemographic questionnaire, recording of physiological parameters, and SAA sampling, will take approximately 15 minutes to complete. The VR headset will be adjusted to the child’s size, and approximately 5 minutes will be allotted for the child to familiarize themselves with the room, equipment, and the game (VR or cartoon on TV or tablet) before the start of the procedure. The intervention will last the entire dental procedure, and its duration will be documented for each patient. DFA assessment by proxy using the VARS will be performed on site by the research assistant present. As per the clinic’s protocol, if a child becomes restless and uncooperative, they will be held by parents for the remainder of the procedure if it cannot be safely stopped at this time or if the procedure is considered an emergency. If the procedure can be safely stopped, rescheduling will be discussed with the parents, including the possible need for sedation. The involvement of parents will be recorded, as will any other distractions used during treatment, such as stickers, teddy bears, music, and others. The occurence of side effects and medications used will be recorded. The former will be transmitted to the ethics committee if they are serious or life-threatening. As VR has the potential to cause nausea, dizziness, and motion sickness, if participants experience any of these, they will be managed in accordance with the clinic’s protocols.

If participants experience severe side effects, the treatment will be safely discontinued. All instruments in the mouth will be removed. The VR device will be removed from the participant. The dental chair will be adjusted to a comfortable position, and the patient will rest until they feel comfortable and recovered. The option to continue the treatment (without the VR headset) or to postpone it to another day will be discussed. Postprocedure SAA sampling and completion of questionnaires on parents’ and health care professionals’ satisfaction levels will take approximately 10 minutes. Equipment used by children will be disinfected and reset before the next participant.

### Statistical Analysis

The primary analyses for this study will follow an intent-to-treat approach, using randomization status as the independent variable, and the mean difference in anxiety (using the anxiety subscale of the Venham) during the dental procedure (T1) and the SAA mean difference between T0 and T2 as the primary outcomes. As there are two primary outcomes (anxiety subscale and SAA), a Bonferroni correction will be applied, and a significance level of *P*=.025 will be used to determine statistical significance of primary outcomes. An ANCOVA, adjusting for unequal variances and adjusted for the time of the procedure, age, and baseline (T0) anxiety or behavioral score, will be used to assess the mean difference in Venham anxiety score during the dental procedure (T1) between the experimental and the control groups. An ANCOVA, adjusted for time of procedure, neurodevelopmental diagnosis, age, and baseline SAA level (T0), will be used to assess the mean difference in SAA level between groups 10 minutes after the procedure (T2).

For the secondary outcomes analyses, parametric tests adjusted for procedural type, age, sex, and parental presence will be used. Comparisons of dichotomous variables, including the necessity of procedural retakes, the use of other nonpharmacological interventions, and the occurrence of side effects, will be assessed using the Cochran-Mantel-Haenszel test. We will also perform subgroup analyses by age group (6‐12 y vs 13‐17 y) and sex and gender (boys, girls, or other) using ANCOVA. Any side effects will be reported using the Medical Dictionary for Regulatory Activities terminology [42], and their proportions will be compared between groups.

### Ethical Considerations

A research assistant will review consent with participants and parents. The information and consent form will be signed by one of the parents on the day of the visit. At enrollment, children are assigned a code used on all data collection forms to protect their confidentiality. Patient identifying information is kept separate from the case reports and is linked to study enrollment by a study ID number. The consent form also identifies all the information that may be collected during this study. All data collected on paper from the day of the intervention will subsequently be transferred into REDCap. No recording of the child’s screen will be collected, nor will their game inputs. As per usual, all information, including links between patient identifiers and study ID, and paper copies of forms and questionnaires, will be stored and double-locked in a cabinet for 7 years (starting from the end of the trial) at the principal investigator’s office at the research center. This trial was approved by the research and ethics board of the Centre Hospitalier Universitaire Sainte-Justine (#2024‐5983). The participants received no compensation.

## Results

This study will be conducted from November 2023 to December 2025. As of November 2025, 300 participants have been recruited. Results are expected to be available in June 2026. The study findings will be disseminated through peer-reviewed publications and presentations at major dental conferences.

## Discussion

### Anticipated Findings

The literature reports that some VR games can induce cybersickness, such as nausea or dizziness associated with computer games, especially in younger individuals with motion sickness susceptibility, or in those who have experienced recent headaches [43]. However, the game used in this study was specifically designed for pediatric patients with reduced speed and eye tracking to minimize these side effects. If the participant feels any symptoms, the game will be stopped and the HMD will be removed. Should the participant experience an injury of any kind following the administration of the study intervention or following any other procedure related to this research, they will receive the appropriate care and services required by their condition.

### Conclusions

The results of the study could be used to better understand how VR distraction reduces dental anxiety and pain in children with SHCN, as well as the satisfaction of parents, patients, and health care professionals with its use during dental procedures. This study may also lead to more comfortable and less traumatic dental care for pediatric patients. In turn, VR can be a valuable tool for managing anxiety in children, which can be a disturbing and anxiety-inducing experience for both parents and health care professionals during dental procedures.

## Supplementary material

10.2196/83672 Multimedia Appendix 1 Parental and participant satisfaction questionnaire.

10.2196/83672 Multimedia Appendix 2 Health care professional satisfaction questionnaire.

10.2196/83672 Multimedia Appendix 3 Face, Legs, Activity, Cry, and Consolability—Revised scale scoring.

10.2196/83672 Checklist 1 SPIRIT Checklist 2025.

## Abbreviations

DFA: dental fear and anxiety
FLACC-R: Face, Legs, Activity, Cry, and Consolability—Revised
HMD: head-mounted device
RCT: randomized controlled trial
REDCap: Research Electronic Data Capture
SAA: salivary alpha-amylase
SHCN: special health care needs
VARS: Venham Anxiety Rating Scale
VR: virtual reality
