# Supplementary material for: Efficacy of a Virtual Reality Game on Children’s Fear and Anxiety During Dental Procedures (VR-TOOTH): Protocol for a Randomized Controlled Trial
Source: JMIR Res Protoc. 2026 Jan 29;15:e83672. doi: 10.2196/83672 (PMC12854657; doi:10.2196/83672)
Supplement: Multimedia Appendix 1 [file resprot-v15-e83672-s001.docx]

**APPENDIX 1**

**Parent/guardian satisfaction**

Considering anxiety relief, side effects, and emotional recovery, how satisfied were you with the intervention used to manage dental fear and anxiety experienced by your child?


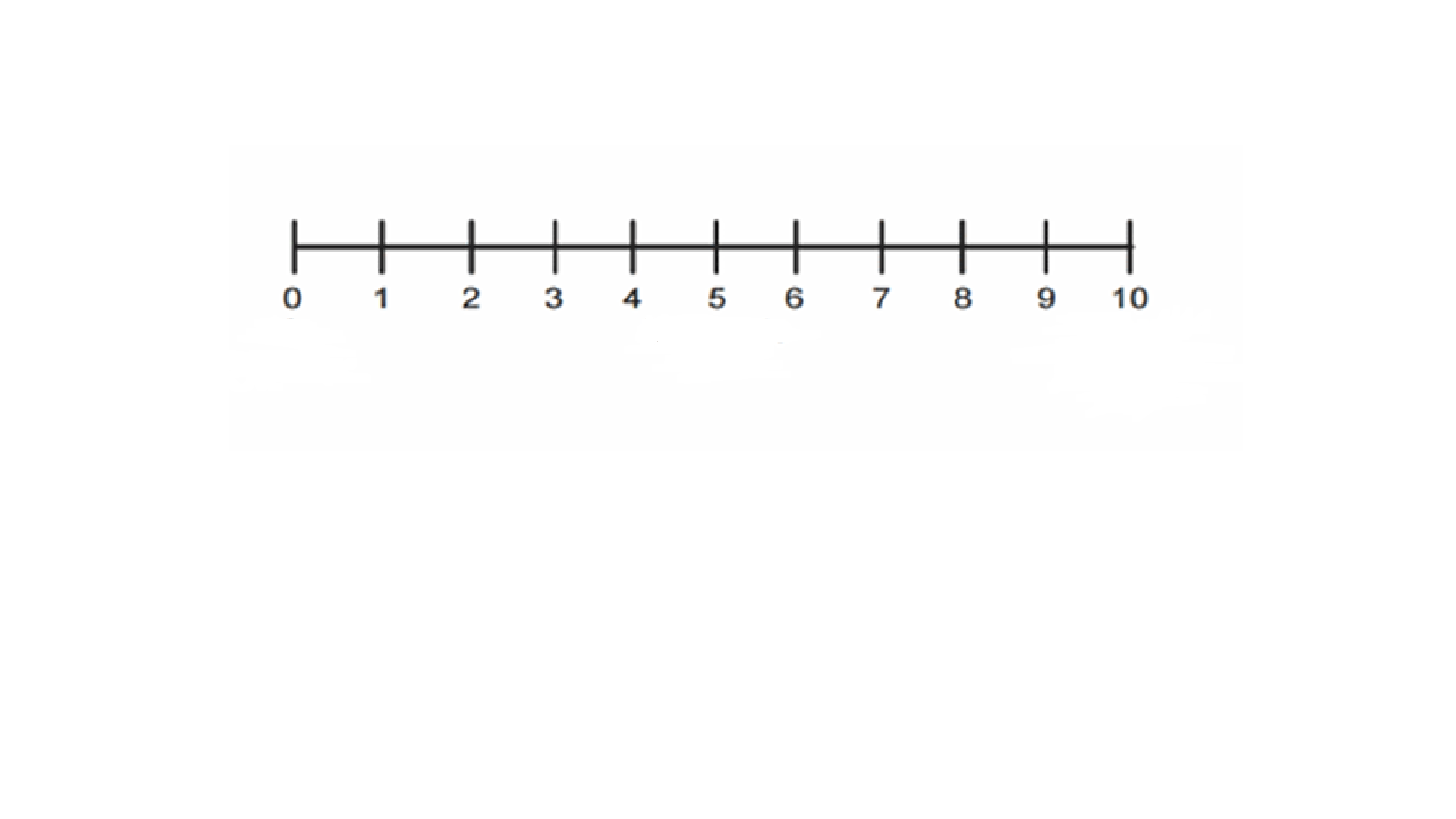


**Very unsatisfied Very satisfied**

Score: ______/10 (self-evaluation)

Do you have any comments? : __________________________________________________________________________________________________________________________________________________________________________________________________________________________________________________________________________________________________________________________________________

**Virtual reality (VR) experience satisfaction scale for patients:**


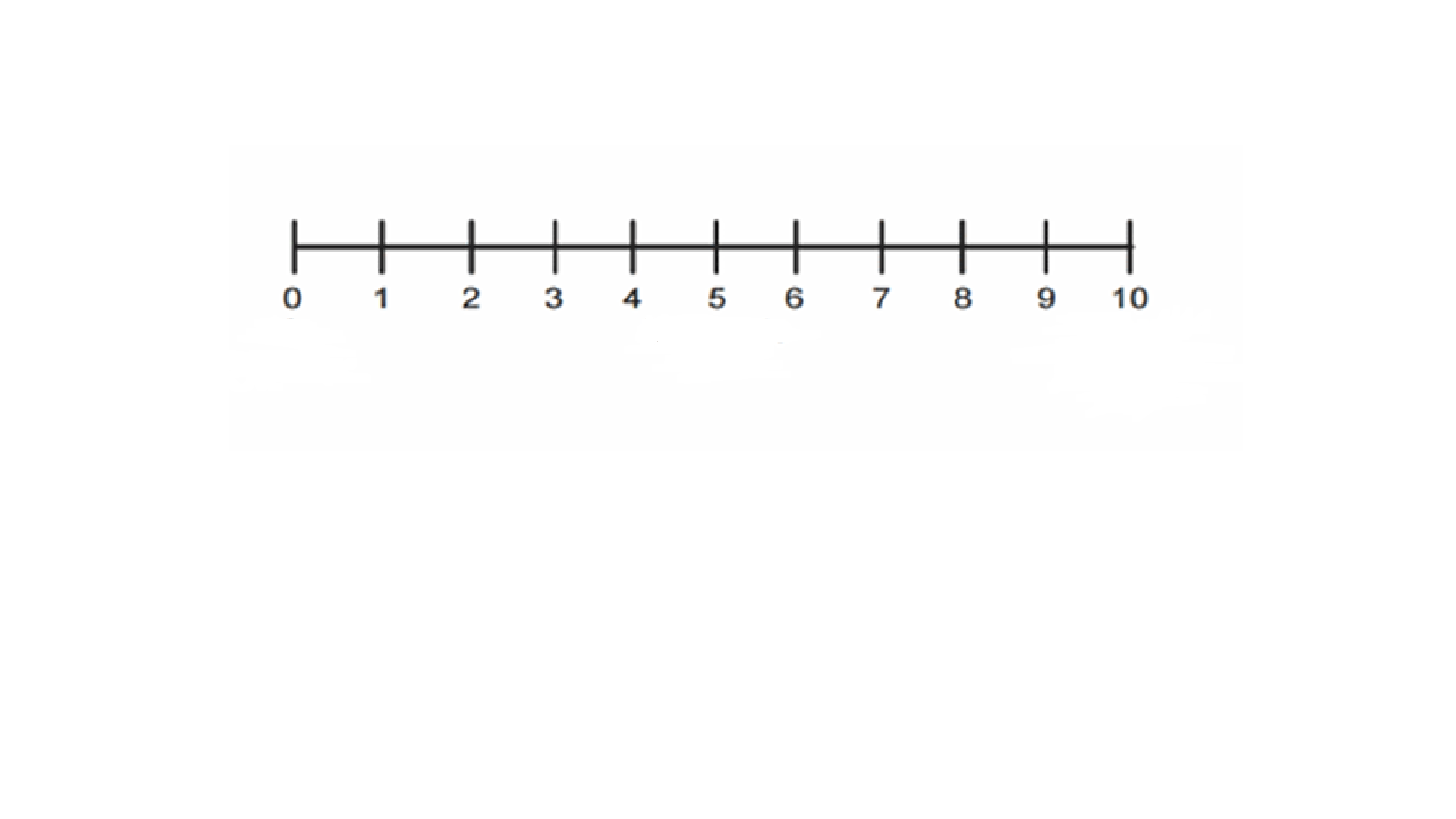


**Very unsatisfied Very satisfied**
