# Supplementary material for: Efficacy of a Virtual Reality Game on Children’s Fear and Anxiety During Dental Procedures (VR-TOOTH): Protocol for a Randomized Controlled Trial
Source: JMIR Res Protoc. 2026 Jan 29;15:e83672. doi: 10.2196/83672 (PMC12854657; doi:10.2196/83672)
Supplement: Multimedia Appendix 2 [file resprot-v15-e83672-s002.docx]

**APPENDIX 2**

| **Research project:** Efficacy of A Virtual Reality Game on Children’s Fear and Anxiety During Dental Procedures (VR-TOOTH): A Randomized Controlled Trial Protocol  You are invited to complete this satisfaction questionnaire if you have performed one or more dental procedures on one or more child(ren)/adolescent(s) who have agreed to participate in the study, **the objective of which is to compare virtual reality with standard care for the management of procedural anxiety related to dental procedures**.  We would like to know your level of satisfaction with these two interventions. For each statement, please check the box that best represents your level of satisfaction. If you have not had the opportunity to use either intervention, please indicate this where applicable.  Participation in this study is free and voluntary. You are therefore free to refuse to complete this satisfaction questionnaire. If you agree to complete this questionnaire, all information collected will remain confidential. By completing this questionnaire, you consent to the study. |
| --- |

| **Healthcare professional satisfaction questionnaire** |
| --- |

**Professional title:**

Dental hygienist

Dental Assistant

Dentist

Dentist in training (Resident)

Other: .............................

**Intervention :**

**What intervention did you just use?**

**Virtual reality ☐ Television ☐**

**Answer the following questions based on the intervention that was used.**

| **Statement** | **Strongly disagree** | **Disagree** | **Agree** | **Strongly agree** |
| --- | --- | --- | --- | --- |
| **1.** Virtual reality/television helped the child control their anxiety/fear |  |  |  |  |
| **2.** Virtual reality/television interfered with the procedure |  |  |  |  |
| **3.** Virtual reality/television helped the child to cooperate better during the procedure |  |  |  |  |
| **4.** I would reuse virtual reality/television for other dental procedures in children |  |  |  |  |
| **5.** The virtual reality/television setup was adapted to the environment of the procedure room. |  |  |  |  |
| **6. For virtual reality only:** The concept of using virtual reality during dental procedures is an idea worth developing. |  |  |  |  |

| **Your preference:**  **Virtual reality**  **Television** |
| --- |

**COMMENTS AND IMPROVEMENTS TO BE MADE:**

**THANK YOU FOR YOUR COOPERATION!**
