# Supplementary material for: Efficacy of a Virtual Reality Game on Children’s Fear and Anxiety During Dental Procedures (VR-TOOTH): Protocol for a Randomized Controlled Trial
Source: JMIR Res Protoc. 2026 Jan 29;15:e83672. doi: 10.2196/83672 (PMC12854657; doi:10.2196/83672)
Supplement: Multimedia Appendix 3 [file resprot-v15-e83672-s003.docx]

**Appendix 3**

| FLACC - Revised Scale Scoring | | | |
| --- | --- | --- | --- |
| Categories | 0 | 1 | 2 |
| Face | No particular expressions or smile. | Occasional grimace or frown, withdrawn, disinterested, sad, appears worried. | Frequent to constant quivering chin, clenched jaw, distressed looking face, expression of fright/ panic. |
| Legs | Normal position or relaxed; usual tone, and motion to limbs. | Uneasy, restless, tense, occasional tremors. | Kicking, or legs drawn up, marked increase in spasticity, constant tremors, jerking. |
| Activity | Lying quietly, normal position, moves easily, regular, rhythmic respirations. | Squirming, shifting back and forth, tense, tense/guarded movements, mildly agitated, shallow/ splinting respirations, intermittent sighs | Arched, rigid or jerking, severe agitation, head banging, shivering, breath holding, gasping, severe splinting. |
| Cry | No cry (awake or asleep) | Moans or whimpers: occasional complaint, occasional verbal outbursts, constant grunting | Crying steadily, screams or sobs, frequent complaints, repeated outbursts, constant grunting. |
| Consolability | Content, relaxed | Reassured by occasional touching, hugging, or being talked to distractible | Difficult to console or comfort, pushing caregiver away, resisting care or comfort measures. |
| Each of the five categories is scored from 0-2, which results in a total score between zero and ten. | | | |
